# Machine-learning model for 30-day mortality in sepsis-associated delirium patients: A retrospective MIMIC-IV cohort study

**DOI:** 10.1097/MD.0000000000045440

**Published:** 2026-01-02

**Authors:** Jingjing Yin, Xuming Pan, Danlei Chen, Jiancheng Zhang, Guangjun Jin

**Affiliations:** a Department of Emergency, The Second Affiliated Hospital of Zhejiang Chinese Medical University, Hangzhou, Zhejiang, China.

**Keywords:** machine learning, MIMIC-IV database, mortality, predictive model, sepsis-associated delirium

## Abstract

Patients with sepsis in the intensive care unit (ICU) are particularly vulnerable to the onset of sepsis-associated delirium (SAD), which is associated with an increased mortality rate. This retrospective cohort study employed machine-learning algorithms to develop a risk-prediction model for 30-day mortality in ICU patients with SAD. Patients with SAD in ICU were extracted from the MIMIC-IV database. Patients were classified into 2 groups: those who survived and those who did not, based on 30-day mortality following ICU admission. The patient data included in this study were subsequently divided into the training and validation sets. The Boruta algorithm was used to identify significant feature indicators. Predictive models have been developed, including logistic regression, support vector machines, decision trees, random forests, extreme gradient boosting, k-nearest neighbors, and naive Bayes. The performance of these models was assessed using a validation set. The final machine-learning model incorporated the Shapley additive explanation method (SHAP) to enhance the interpretability of predictive outcomes. In total, 5390 patients were diagnosed with SAD using the MIMIC-IV database. The XGBoost model exhibited the highest predictive accuracy and was chosen as the final model, achieving an area under the receiver operating characteristic curve of 0.743 for the validation set. Using the SHAP method, the top 15 significant features were identified in the XGBoost predictive model. The SHAP analysis identified blood urea nitrogen, age, prothrombin time, partial thromboplastin time, and history of stroke as the top predictors of mortality. The XGBoost model demonstrated superior performance in forecasting 30-day mortality among ICU patients with SAD. In contrast to conventional predictive models, this machine-learning approach enables the prediction of 30-day mortality within 24 h of patient’s admission. However, the model’s low specificity may limit its clinical utility, and external validation is needed.

## 1. Introduction

Delirium is an acute cognitive disturbance characterized by variations in consciousness, a lack of attention, cognitive impairment, and perceptual anomalies. Sepsis-associated delirium (SAD) frequently occurs in the Intensive Care Unit (ICU) and is significantly associated with increased mortality and unfavorable outcomes.^[1]^ Studies have shown that individuals diagnosed with SAD experience an increased mortality rate and extended stay in the ICU.^[2]^

Delirium is a prevalent and significant complication in the ICU, with various assessment tools available, the most commonly utilized being the Confusion Assessment Method for the ICU (CAM-ICU). The CAM-ICU is widely implemented in ICUs around the globe due to its versatility and efficacy across diverse cultural and linguistic contexts. A multinational, multicenter study revealed that the incidence of delirium in patients evaluated with the CAM-ICU was closely associated with sedation levels, particularly at higher sedation levels, which led to more precise outcomes in CAM-ICU assessments.^[3]^ Furthermore, a separate study suggests that the application of the CAM-ICU assessment tool, along with nurse training, can markedly diminish the reliance on high-risk medications, consequently lowering the occurrence of delirium.^[4]^ Finally, the CAM-ICU has shown to be more effective than other assessment instruments. A study comparing the CAM-ICU with the Intensive Care Delirium Screening Checklist (ICDSC) revealed that the CAM-ICU had a stronger association with neurological deficits in the evaluation of delirium among neurocritical care patients, indicating its potential utility in targeted patient groups.^[5]^

Conventional prognostic models for patients with delirium generally depend on clinical scoring systems, including Acute Physiology and Chronic Health Evaluation (APACHE) and Sequential Organ Failure Assessment (SOFA). Nonetheless, these models exhibit specific limitations regarding their predictive accuracy and ability to provide personalized predictions.^[6]^ Traditional models may fail to sufficiently consider individual patient variations and intricate pathophysiological alterations, leading to reduced accuracy in predicting outcomes.^[7]^

In contrast, predictive models based on machine learning offer considerable benefits in managing intricate datasets and identifying potential risk factors. By leveraging electronic health records (EHR) along with diverse machine-learning algorithms, these models can enhance the accuracy of predictions regarding the onset and prognosis of SAD.^[8,9]^ For example, machine-learning techniques such as Random Forest (RF) and Extreme Gradient Boosting (XGBoost) have demonstrated outstanding efficacy in forecasting SAD, achieving high levels of accuracy and sensitivity.^[2,10]^ Furthermore, machine-learning models can deliver individualized risk assessments and predictions by incorporating a range of clinical variables including blood urea nitrogen levels, respiratory rate, and urine output.^[11]^ These models not only improve prediction accuracy but also aid clinicians in comprehending the rationale behind the model’s predictions through interpretability analyses, such as Shapley additive explanation method (SHAP) analysis, thus facilitating more informed clinical decision-making.^[12]^ Currently, there is a lack of research focused on employing machine-learning techniques to forecast outcomes in individuals with SAD.

This study sought to use extensive clinical data from the MIMIC-IV database. By gathering clinical information from patients with SAD at the time of ICU admission, we aimed to create a machine-learning model that identifies risk factors linked to mortality within 30 days of ICU admission. The primary objective of this study was to develop and validate a machine-learning model for predicting 30-day mortality in ICU patients with SAD.

## 2. Methods

### 2.1. Study population

This study focused on patients with sepsis-related delirium who were admitted to the ICU, as documented in the Medical Information Mart for Intensive Care IV (MIMIV-IV) database. The inclusion criteria were individuals aged ≥ 18 years and those who had an ICU stay of at least 24 hours. Exclusion criteria included patients with multiple ICU readmissions, those who exhibited delirium prior to the onset of sepsis, individuals who experienced delirium during non-ICU hospitalizations, patients who were unable to undergo delirium assessments or lacked any records of such assessments, and those with negative Confusion Assessment Method for the ICU (CAM-ICU) results. Sepsis was diagnosed according to the “Sepsis-3.0” criteria, which classifies sepsis as a SOFA score of 2 or more points in conjunction with an infection or suspected infection. Suspected infection was indicated by the initiation of antibiotic therapy within 3 days or 24 h following specimen collection. Sepsis-related delirium was identified through positive Confusion Assessment Method-ICU (CAM-ICU) screening after the diagnosis of sepsis.

### 2.2. Study design

The primary objective was to develop and validate a machine-learning model predicting 30-day mortality in ICU patients with SAD. This was a retrospective observational study in which the research team extracted data from patients with SAD from the MIMIC-IV (version 3.1) database. The MIMIC-IV database contains information on all patients admitted to the Beth Israel Deaconess Medical Center (BIDMC) between 2008 and 2022. It includes extensive data on each patient’s length of stay, laboratory tests, medication management, vital signs, and additional clinical information. To ensure patient confidentiality, all personal identifiers were de-identified and random codes were assigned in lieu of patient identifiers. Consequently, this study did not require any patient consent or ethical approval. The authors have completed the Collaborative Institutional Training Initiative (CITI) certification (certificate number: 47386519) and have successfully gained access to the database.

### 2.3. Data extraction and processing

Data obtained from the MIMIC-IV database for the patients involved in this study included the following components: (1) demographic characteristics of the patients; (2) initial vital signs recorded within the first 24 hours of ICU admission, encompassing temperature, heart rate, respiratory rate, systolic blood pressure, diastolic blood pressure, mean arterial pressure, and oxygen saturation; (3) laboratory test results collected within 24 hours of ICU admission, which included white blood cell count, platelet count, hemoglobin levels, blood urea nitrogen, serum creatinine, potassium, sodium, chloride, calcium, phosphorus, magnesium, glucose, prothrombin time (PT), activated partial thromboplastin time (PTT), international normalized ratio, anion gap, and bicarbonate concentration; (4) Glasgow Coma Scale (GCS) scores and SOFA scores recorded within the first 24 hours of ICU admission; (5) comorbid conditions (acute myocardial infarction, chronic kidney disease, chronic obstructive pulmonary disease, hypertension, diabetes, acute kidney injury, and stroke); (6) details on the administration of mechanical ventilation, renal replacement therapy, vasoactive medications, and sedatives within 24 hours of ICU admission; (7) patient survival duration and mortality rates within 30 days following ICU admission; (8) delirium data extracted from the “chartevents” table within the “mimic_icu” module of the MIMIC-IV database, identified by the item ID “228332.”

### 2.4. Approaches for developing machine-learning models

Patient data from the MIMIC-IV database were randomly allocated to training and validation sets in a 7:3 ratio. Following the selection of clinical feature variables using the Boruta algorithm, machine-learning predictive models were developed using the R software (version 4.4.3). The “base” package was utilized for constructing the logistic regression (LR) model, the “e1071” package was employed for the support vector machine (SVM) and naive Bayes (NB) models, the “xgboost” package was used for the extreme gradient boosting (XGBoost) model, the “randomForest” package was applied for the RF model, the “rpart” package was utilized for the decision tree (DT) model, and the “kknn” package was employed for the k-nearest neighbors (KNN) model.

### 2.5. Statistical analysis

Continuous variables are expressed as medians and interquartile ranges. The Mann–Whitney U test was used for statistical comparisons between the 2 groups. Categorical variables were presented as counts and percentages, and the chi-square test or Fisher exact test was used for group comparisons. Receiver operating characteristic (ROC) curves were generated for both the LR and machine-learning models. The area under the ROC curve (AUROC), specificity, sensitivity, positive predictive value, negative predictive value, accuracy, and kappa coefficient were used to assess model performance, with AUROC being the primary metric of interest. The model that demonstrated the highest predictive performance was designated the principal model for this study. Calibration curves were used to evaluate the alignment between the observed and predicted outcomes, whereas decision curve analysis was performed to determine the net clinical benefit. Additionally, the SHAP method was applied to enhance the interpretability of the final predictive model, where higher SHAP values indicated a greater likelihood of delirium development. All statistical analyses were conducted using the R software (version 4.4.3), with a significance threshold set at *P* < .05.

## 3. Results

### 3.1. Baseline characteristics of SAD patients

**Figure 1.**
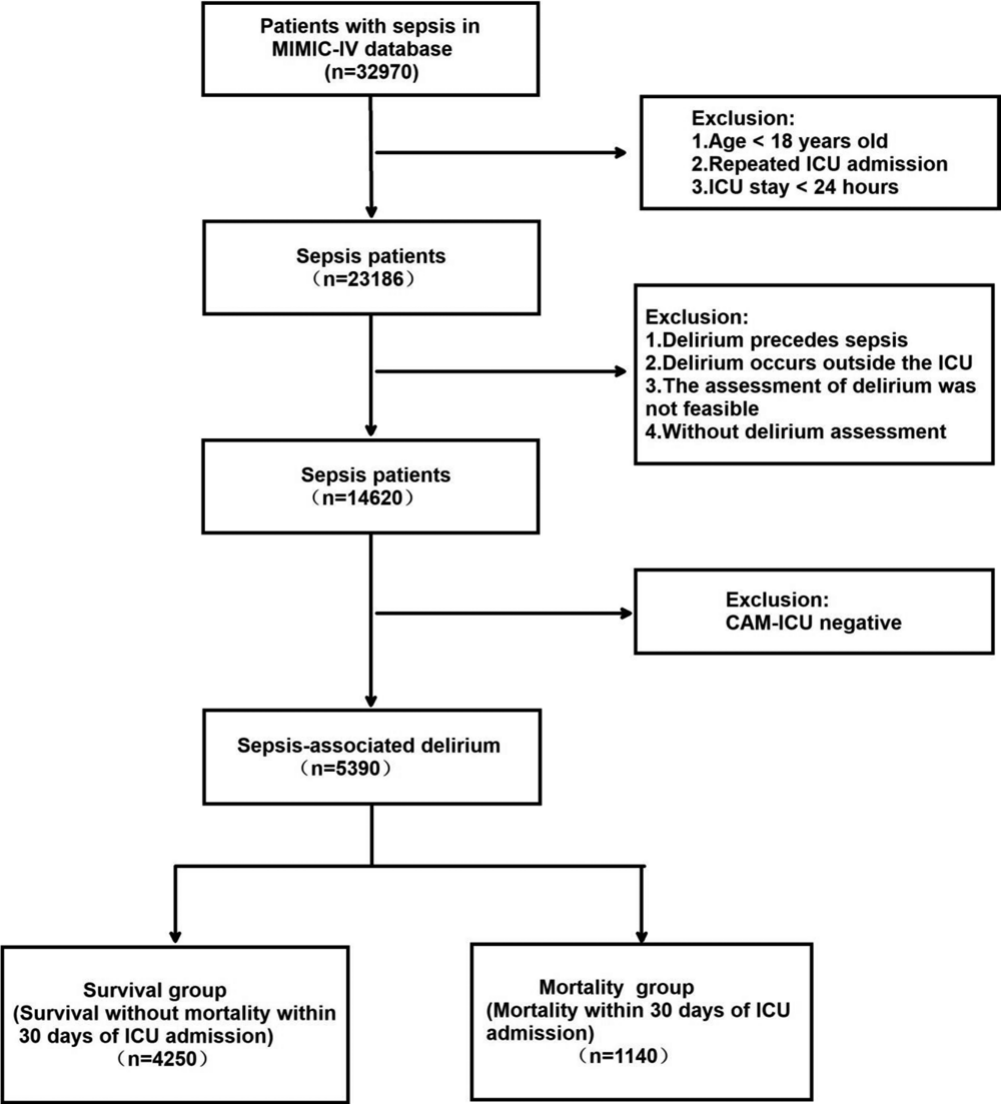
Process flowchart for determining inclusion and exclusion criteria for this study.

Based on the established inclusion and exclusion criteria, this study analyzed 5390 patients with SAD from the MIMIC-IV database, as depicted in Figure 1. The patients were categorized into survival and mortality groups depending on whether they succumbed within 30 days. A comparison of baseline characteristics between the 2 groups is presented in Table 1. In this study 1140 patients (21.2%) died within 30-day period. Analysis of the data revealed that the mortality group was older, exhibited a lower mean arterial pressure, demonstrated impaired renal and coagulation functions, had a higher incidence of vasopressor utilization, and presented elevated SOFA scores. Furthermore, the prevalences of hypertension, acute myocardial infarction, chronic kidney disease, acute kidney injury, and stroke were significantly higher in the mortality group. The differences between the 2 groups were statistically significant (*P* < .05).

**Table 1:** Baseline characteristics of survival group and mortality group.

Variables	MIMIC-IV cohort		*P*
	Survival group (n = 4250)	Mortality group (n = 1140)	
Age	68.00 [56.00, 78.00]	74.00 [63.00, 83.00]	<.001
Weight (kg)	80.30 [67.30, 97.00]	76.50 [64.00, 93.17]	<.001
Sex, n (%)			
Female	2494 (58.7)	608 (53.3)	.001
Male	1756 (41.3)	532 (46.7)	
Vital signs			
Temperature (°C)	36.83 [36.50, 37.22]	36.72 [36.44, 37.11]	<.001
Heart rate (min^−1^)	89.00 [77.00, 105.00]	93.00 [80.00, 109.00]	<.001
Respiratory rate (min^−1^)	20.00 [16.00, 24.00]	21.00 [17.00, 25.00]	<.001
SpO2 (%)	98.00 [95.00, 100.00]	97.00 [94.00, 100.00]	<.001
Systolic BP (mm Hg)	121.00 [104.00, 139.00]	116.00 [100.00, 133.00]	<.001
Diastolic BP (mm Hg)	66.00 [55.00, 79.00]	62.50 [52.00, 76.00]	<.001
Mean arterial BP (mm Hg)	82.00 [70.00, 94.00]	77.00 [67.00, 89.00]	<.001
Laboratory tests			
WBC (k/µL)	12.10 [8.40, 16.60]	12.60 [8.70, 18.53]	.004
Hemoglobin (g/dL)	10.50 [8.80, 12.20]	10.00 [8.40, 11.60]	<.001
Platelet (k/µL)	177.00 [124.00, 240.00]	176.00 [104.00, 252.25]	.132
BUN (mg/dL)	21.00 [14.00, 35.00]	32.00 [20.00, 51.00]	<.001
Creatinine (mg/dL)	1.10 [0.80, 1.70]	1.40 [0.90, 2.20]	<.001
Glucose (mg/dL)	137.50 [110.00, 178.00]	138.00 [107.00, 184.00]	.522
Sodium (mEq/L)	138.00 [135.00, 141.00]	138.00 [134.00, 142.00]	.641
Chloride (mEq/L)	104.00 [100.00, 108.00]	103.00 [98.00, 108.00]	<.001
Potassium (mEq/L)	4.10 [3.70, 4.70]	4.30 [3.80, 4.80]	.002
Magnesium (mg/dL)	1.90 [1.70, 2.20]	2.00 [1.80, 2.30]	<.001
Total calcium (mg/dL)	8.20 [7.70, 8.70]	8.20 [7.60, 8.80]	.412
Phosphate (mg/dL)	3.60 [2.90, 4.50]	4.00 [3.10, 5.20]	<.001
INR	1.30 [1.10, 1.60]	1.45 [1.20, 2.00]	<.001
Prothrombin time (s)	14.10 [12.40, 17.20]	15.70 [13.00, 21.63]	<.001
PTT (s)	30.80 [27.20, 37.40]	33.80 [28.40, 45.40]	<.001
Bicarbonate (mEq/L)	22.00 [19.00, 25.00]	21.00 [18.00, 24.00]	<.001
Anion gap (mmol/L)	15.00 [13.00, 18.00]	16.00 [14.00, 20.00]	<.001
Score			
GCS	15.00 [15.00, 15.00]	15.00 [14.00, 15.00]	.482
SOFA	3.00 [2.00, 5.00]	4.00 [2.00, 5.00]	<.001
Treatment measures			
MV, n (%)	2645 (62.2)	696 (61.1)	.486
CRRT, n (%)	117 (2.8)	61 (5.4)	<.001
Vasopressor, n (%)	2207 (51.9)	693 (60.8)	<.001
Sedation, n (%)	1947 (45.8)	449 (39.4)	<.001
Comorbidity			
AMI, n (%)	510 (12.0)	197 (17.3)	<.001
COPD, n (%)	196 (4.6)	50 (4.4)	.807
Hypertension, n (%)	1795 (42.2)	431 (37.8)	.008
Diabetes, n (%)	720 (16.9)	185 (16.2)	.598
Stroke, n (%)	502 (11.8)	186 (16.3)	<.001
CKD, n (%)	827 (19.5)	300 (26.3)	<.001

Continuous variables were expressed as median and interquartile range, the Mann–Whitney *U* test was used for statistical comparisons between 2 groups. Categorical variables were described as counts and percentages, and the Chi-squared test or Fisher exact test was used for group comparisons.

AMI = acute myocardial infarction, BP = blood pressure, BUN = blood urea nitrogen, CKD = chronic kidney disease, COPD = chronic obstructive pulmonary disease, CRRT = continuous renal replacement therapy, GCS = Glasgow coma scale, INR = international normalized ratio, MV = mechanical ventilation, PTT = partial thromboplastin time, SOFA = sequential organ failure assessment, WBC = white blood cell count.

### 3.2. Selection of clinical characteristic features

**Figure 2.**
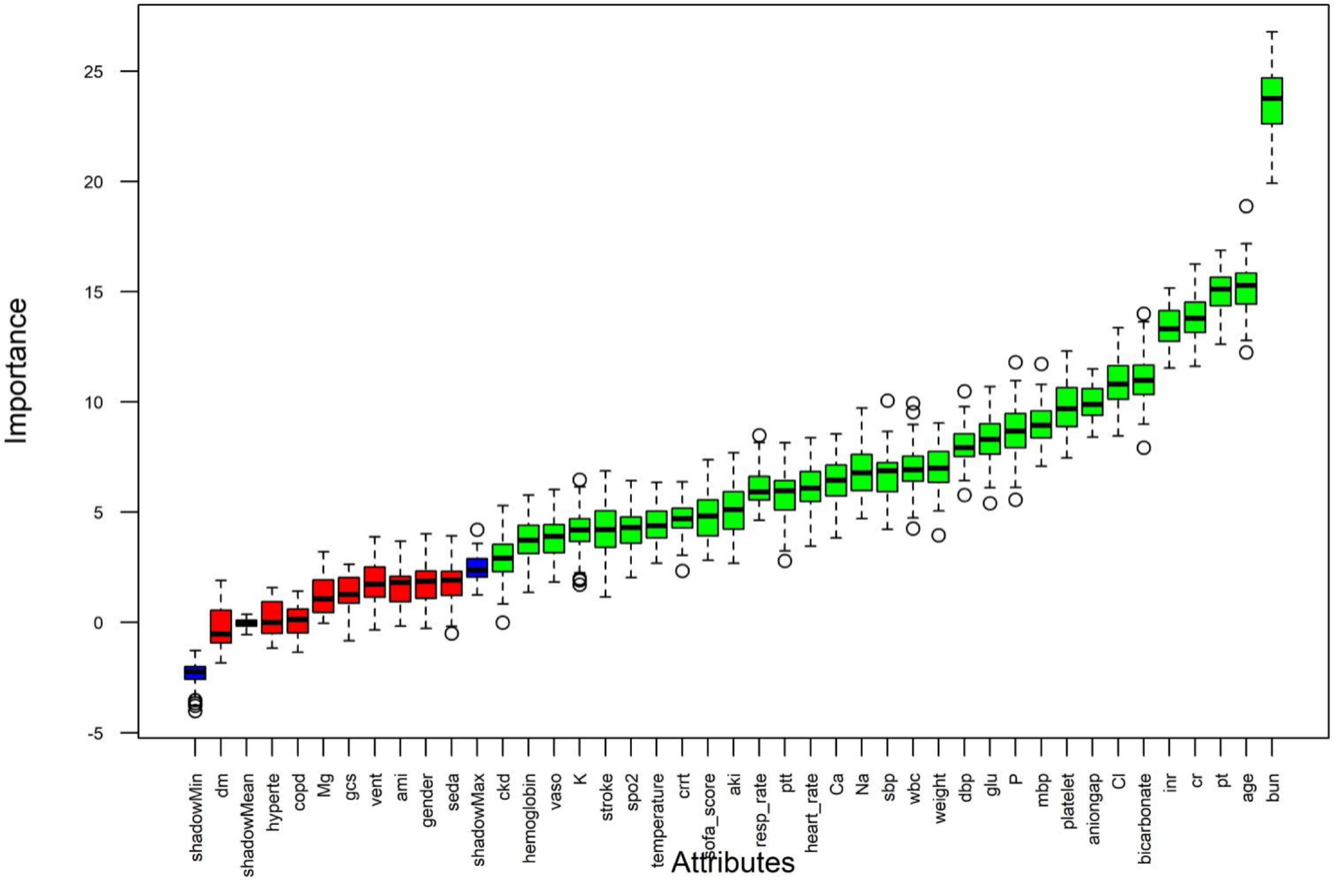
Selection of clinical feature variables using the Boruta algorithm. aki = acute kidney injury, ami = acute myocardial infarction, aniongap = anion gap, bun = blood urea nitrogen, Ca = total calcium, ckd = chronic kidney disease, Cl = chloride, cr =creatinine, crrt = continuous renal replacement therapy, dbp = diastolic blood pressure, dm = diabetes, gcs = Glasgow Coma Scale, glu = glucose, hyperte = hypertension, K = potassium, mbp = mean arterial blood pressure, Mg = magnesium, Na = sodium, P = phosphate, pt = prothrombin time, resp_rate = respiratory rate, sbp = systolic blood pressure, seda = sedation, vaso = vasopressor, vent = mechanical ventilation, wbc = white blood cell count.

Prior to the development of the machine-learning model, 40 clinical feature variables were identified (Table 1). The Boruta algorithm was subsequently employed to filter these variables, yielding 31 clinical features that exhibited the strongest association with 30-day mortality in patients ranked according to their Z-scores (Table 2). The variables included blood urea nitrogen, age, PT, creatinine, INR, bicarbonate, chloride, anion gap, platelet, mean arterial blood pressure, serum phosphorus, glucose, diastolic blood pressure, weight, white blood cell count, systolic blood pressure, sodium, total calcium, heart rate, PTT, respiratory rate, acute kidney injury, SOFA score, continuous renal replacement therapy, temperature, SpO_2_, stroke, potassium, vasopressor, hemoglobin, and chronic kidney disease. The Z-scores for these clinical feature variables were significantly greater than the maximum Z-score of the shadow features, underscoring their substantial influence on the outcome, as illustrated in green in Figure 2.

**Table 2:** Boruta mean *Z*-score for all variables.

Variable	Mean *Z*-score	Decision	Variable	Mean *Z*-score	Decision
bun	25.323	Confirmed	resp_rate	5.030	Confirmed
age	15.899	Confirmed	K	4.504	Confirmed
pt	14.862	Confirmed	crrt	4.188	Confirmed
cr	14.799	Confirmed	sofa_score	3.918	Confirmed
bicarbonate	12.865	Confirmed	vaso	3.903	Confirmed
inr	12.818	Confirmed	temperature	3.771	Confirmed
Cl	10.625	Confirmed	spo2	3.587	Confirmed
platelet	10.574	Confirmed	hemoglobin	3.537	Confirmed
aniongap	9.678	Confirmed	ckd	3.080	Confirmed
mbp	8.932	Confirmed	shadowMax	2.119	NA
glu	8.645	Confirmed	gender	1.870	Rejected
P	8.369	Confirmed	shadowMean	−0.036	NA
Ca	7.964	Confirmed	shadowMin	−1.791	NA
dbp	7.931	Confirmed	Mg	−Inf	Rejected
wbc	7.506	Confirmed	gcs	−Inf	Rejected
weight	7.308	Confirmed	vent	−Inf	Rejected
sbp	6.744	Confirmed	seda	−Inf	Rejected
heart_rate	6.656	Confirmed	ami	−Inf	Rejected
Na	6.169	Confirmed	copd	−Inf	Rejected
aki	6.044	Confirmed	hyperte	−Inf	Rejected
ptt	5.647	Confirmed	dm	−Inf	Rejected
stroke	5.241	Confirmed			

Variables with *Z*-score = −Inf were considered completely uninformative and thus excluded from further analysis.

aki = acute kidney injury, ami = acute myocardial infarction, aniongap = anion gap, bun = blood urea nitrogen, Ca = total calcium, ckd = chronic kidney disease, Cl = chloride, cr = creatinine, crrt = continuous renal replacement therapy, dbp = diastolic blood pressure, dm = diabetes, gcs = Glasgow coma scale, glu = glucose, hyperte = hypertension, K = potassium, mbp = mean arterial blood pressure, Mg = magnesium, Na = sodium, P = phosphate, pt = prothrombin time, resp_rate = respiratory rate, sbp = systolic blood pressure, seda = sedation, vaso = vasopressor, vent = mechanical ventilation, wbc = white blood cell count.

### 3.3. Development of machine-learning models and evaluation of their performance

The collected data were partitioned into training and validation sets in a ratio of 7:3. Utilizing the clinical features identified by the Boruta algorithm, we incorporated 31 clinical feature variables to develop machine-learning models. We employed suitable R packages to construct a traditional LR model along with 6 machine-learning models: SVM, Extreme Gradient Boosting (XGBoost), RF, DT, NB, and K-Nearest Neighbors (KNN).

**Figure 3.**
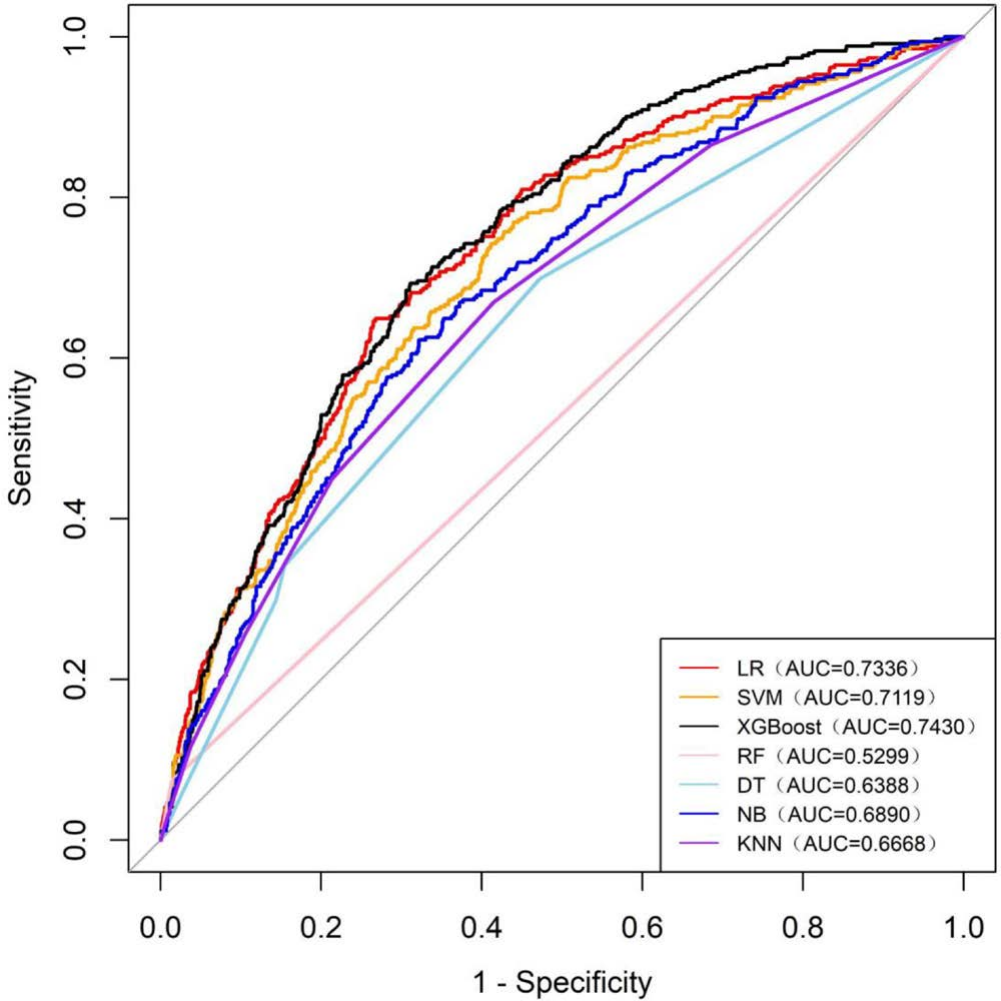
The receiver operating characteristic (ROC) curves of the LR, SVM, XGBoost, RF, DT, NB, and KNN models on the validation set. DT = decision tree model, KNN = K-nearest neighbors model, LR = logistic regression model, NB = naive Bayes model, NPV = negative predictive value, PPV = positive predictive value, RF = random forest model, SVM = support vector machine model, XGBoost = extreme gradient boosting model.

Table 3 presents the predictive performances of the models evaluated using the validation set. The XGBoost model demonstrated the highest predictive accuracy, achieving an area under the ROC curve (AUROC) of 0.743, which reflects a moderate level of predictive capability. This was closely followed by the LR model, which recorded an AUROC of 0.734, and the SVM model, which recorded an AUROC of 0.712. The other models exhibited AUROCs < 0.7, indicating limited predictive effectiveness (Fig. 3).

**Table 3:** Model performance on the validation set.

Model	AUROC	Sensitivity	Specificity	PPV	NPV	Accuracy	Kappa
LR	0.734	0.969	0.146	0.809	0.562	0.795	0.159
SVM	0.712	0.970	0.117	0.804	0.513	0.790	0.122
XGBoost	0.743	0.965	0.117	0.803	0.471	0.785	0.113
RF	0.530	0.987	0.073	0.799	0.595	0.793	0.088
DT	0.639	0.973	0.059	0.794	0.370	0.780	0.046
NB	0.689	0.850	0.357	0.831	0.390	0.746	0.214
KNN	0.667	0.988	0.038	0.793	0.464	0.787	0.040

DT = decision tree model, KNN = K-nearest neighbors model, LR = logistic regression model, NB = naive Bayes model, NPV = negative predictive value, PPV = positive predictive value, RF = random forest model, SVM = support vector machine model, XGBoost = extreme gradient boosting model.

**Figure 4.**
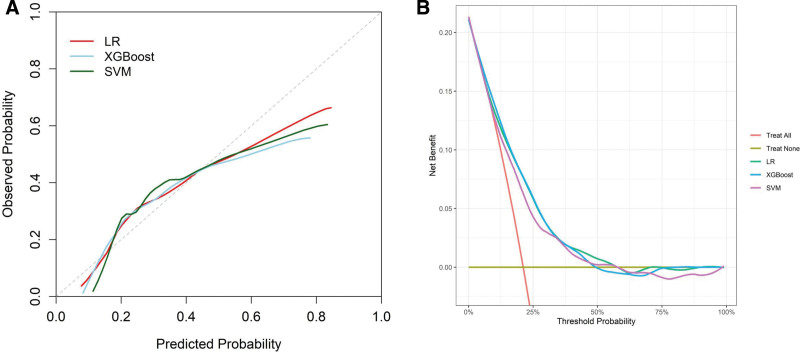
(A) Calibration curves of the LR, XGBoost, SVM models. (B) Decision curves of the LR, XGBoost, SVM models. LR = logistic regression model, SVM = support vector machine model, XGBoost = extreme gradient boosting model.

To assess the calibration of the models, we generated and compared calibration curves for the 3 top-performing models: XGBoost, LR, and SVM (Fig. 4A). Among these models, XGBoost displayed the most accurate alignment between observed and predicted probabilities, signifying its superior calibration. Additionally, we performed decision curve analysis for these 3 models, with the findings illustrated in Figure 4B. The analysis revealed that the XGBoost predictive model yielded the greatest net benefit for forecasting 30-day mortality in patients with SAD, surpassing the performances of both the LR and SVM models.

### 3.4. Model interpretations

**Figure 5.**
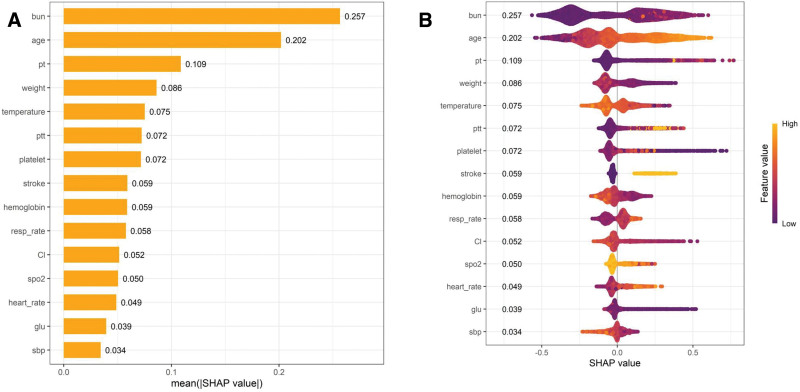
(A) Feature importance ranking plot of the XGBoost model (top 15 features). (B) SHAP summary plot of the XGBoost model (top 15 features). bun = blood urea nitrogen, Cl = chloride, glu = glucose, pt = prothrombin time, ptt = partial thromboplastin time, resp_rate = respiratory rate, sbp = systolic blood pressure.

The opaque nature of machine-learning models often complicates the task of offering comprehensive explanations of the variables incorporated within these models. To assess the significance of the variables in the XGBoost model, we utilized the Shapley Additive Explanations (SHAP) method to enhance the interpretability of the final predictive model. Elevated SHAP values suggested a greater probability of mortality within 30 days in patients with SAD. We present the feature importance ranking derived from the XGBoost model, highlighting the top 15 features (Fig. 5A). These features included blood urea nitrogen, age, PT, weight, body temperature, PTT, platelets, stroke, hemoglobin, respiratory rate, chloride, SpO2, heart rate, glucose, and systolic blood pressure. Additionally, the SHAP summary beeswarm plot (Fig. 5B) further elucidates the ranking of these features by showing their respective impacts on the model’s output. Each point in the plot represents the SHAP value of a feature in a specific case. The y-axis denotes the features, whereas the x-axis reflects the SHAP value, indicating the extent of each feature’s influence on the prediction. The colors of the points correspond to the actual values of the features, with purple representing lower values and yellow indicating higher values. For instance, in the case of age, the yellow points positioned to the right of the zero line signify that elevated values are associated with an increased risk of 30-day mortality.

**Figure 6.**
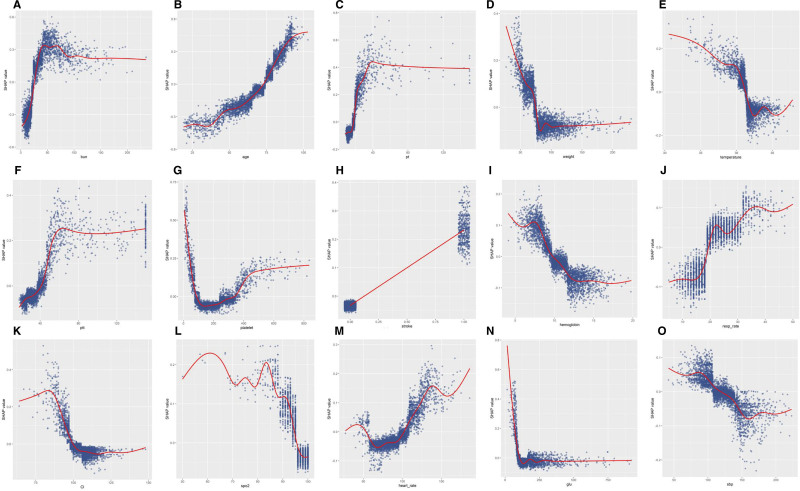
(A–O): Partial dependence plots of features. Y-axis represents SHAP values; X-axis represents actual clinical parameters for continuous variables, and for binary variables (e.g., stroke), “0” indicates absence and ‘1’ indicates presence of the condition. bun = blood urea nitrogen, Cl = chloride, glu = glucose, pt = prothrombin time, ptt = partial thromboplastin time, resp_rate = respiratory rate, sbp = systolic blood pressure.

Partial dependence plots (PDPs) visually represent the marginal impacts of various clinical feature variables on the predictions generated by the machine-learning models (Fig. 6). In these plots, the x-axis denotes the actual values of the clinical parameters, whereas the y-axis represents the corresponding SHAP values. This approach allows the quantification of the relationship between features and risk. A significant benefit of PDPs is their capacity to reveal nonlinear relationships between features and outcomes. When the plotted lines exhibit curvature or directional changes, they signify a nonlinear association between the feature and the outcome. Thus, PDPs provide a more intricate understanding of a model’s decision-making processes, extending beyond the insights offered by linear models. For binary features such as stroke, the 2 distinct states of the variable are represented along the x-axis. The y-axis reflects the average predicted outcome for instances corresponding to each state. If the average predicted value for 1 state exceeds that of the other, it implies that this state is more likely to result in the predicted outcome. It is crucial to recognize that curve fitting for binary variables in PDPs does not indicate trends or gradients as it does for continuous variables; rather, it merely connects the average predicted values of the 2 states.

## 4. Discussion

The 30-day mortality rate of patients experiencing sepsis-related delirium in this study was 21.2%, which is consistent with the findings reported by Shen.^[13]^ In this study, among the 6 machine-learning models developed, XGBoost exhibited the highest diagnostic performance. Both the calibration curve and calibration plot demonstrated that XGBoost delivered the best fit and a net benefit. XGBoost is an ensemble machine-learning algorithm grounded in DTs that operates within the framework of gradient boosting.^[14]^ While artificial neural networks typically excel in predictive tasks involving unstructured data, such as images and text, DT-based algorithms are increasingly recognized as the superior choice for medium to small structured or tabular datasets. XGBoost has been noted for its efficiency, flexibility, and lightweight design, making it widely applicable in areas such as data mining and recommendation systems. In the context of clinical predictive models, the XGBoost model presents several advantages^[7]^: high accuracy: XGBoost is proficient in addressing classification and regression tasks, effectively capturing nonlinear relationships and interactions within the data; robust feature selection: XGBoost ranks feature importance, identifying the most influential features on prediction outcomes, thereby enhancing the model’s interpretability and predictive capability; support for diverse objective functions: XGBoost accommodates various objective functions (including regression, classification, and ranking) and permits customization of loss functions and evaluation metrics to suit different application requirements; automatic missing-value handling: XGBoost manages missing values internally, negating the need for extensive data preprocessing; overfitting mitigation: the model’s complexity is controlled through early stopping techniques and regularization (L1 and L2), minimizing the likelihood of overfitting.

As XGBoost operates as a “black box” model in machine learning, it is challenging to intuitively elucidate the influence of individual clinical features on clinical outcomes, unlike LR models. Consequently, this study utilized the Shapley additive explanations (SHAP) method to offer partial insights into the top 15 most significant clinical features incorporated in the XGBoost model.

Current literature on SAD has largely concentrated on its diagnosis, prevalence, and associated risk factors. For instance, Gu et al^[15]^ identified several predictive indicators for the development of delirium in septic patients, including the SOFA score, necessity for mechanical ventilation, initial lactate levels, and serum phosphate levels at the time of ICU admission. Another investigation highlighted that emergency surgical procedures and elevated doses of midazolam and fentanyl may act as independent risk factors for SAD in patients requiring mechanical ventilation due to sepsis.^[16]^ Furthermore, Zhang^[2]^ revealed that the XGBoost model outperformed other predictive models in forecasting SAD. It is important to note that these studies have primarily focused on the diagnosis of SAD, with a scarcity of research dedicated to predicting its prognosis.

This study demonstrated that BUN levels are a critical determinant of the 30-day mortality rate in patients with SAD, as indicated by the XGBoost model. BUN, a widely recognized biomarker, has been the subject of extensive investigation regarding its predictive capability in critically ill individuals. The literature suggests that increased BUN levels are strongly linked to the development of SAD and may contribute to negative patient outcomes.^[2,17–19]^ A previous study revealed that BUN levels demonstrated a nonlinear association with 28-day mortality. Notably, when BUN levels surpassed a specific threshold, the risk of mortality increased markedly.^[17]^ Furthermore, the BUN-to-serum albumin ratio (BAR) has been employed as a prognostic indicator in patients with sepsis. Research indicates that a higher BAR correlates with an elevated risk of mortality in both the short- and long-term.^[20,21]^ Additional studies have investigated the synergistic effects of BUN and other biomarkers. Notably, the BUN-to-creatinine ratio (BCR) has emerged as a promising predictor of delirium onset in critically ill patients, especially in cases of hypoactive delirium.^[22]^ Additionally, research examining the relationship between BUN and the triglyceride-glucose index (TyG index) indicates that BUN may act as a mediator in these connections, thereby affecting mortality risk among patients.^[23]^ In summary, BUN is a readily accessible biomarker with considerable clinical utility in the ICU. By tracking BUN levels, healthcare professionals can more effectively identify patients at a high risk for SAD, facilitating timely interventions that can enhance patient outcomes.

In this study, age was identified as a critical risk factor. It is widely recognized that with advancing age, numerous physiological functions progressively deteriorate. Consequently, older patients exhibit increased mortality rates and heightened risk of negative outcomes.^[24,25]^ The incidence of delirium in the ICU is significantly associated with age, especially in older patients, where it is more common.^[26]^ The results of this study suggest that the likelihood of developing delirium increases considerably with advancing age, underscoring the importance of prioritizing the prevention and management of delirium in older patients in clinical settings. A study examining elderly individuals with sepsis revealed a significant association between age and mortality rates at both 28 and 90 days, especially among patients aged over 80 years, who exhibited substantially higher mortality rates than their younger counterparts.^[25]^ Additionally, a separate study revealed that older patients exhibit an elevated mortality rate in the ICU, which increases with advancing age.^[24]^ This finding implies that age serves as an independent prognostic factor in patients with SAD and may interact with other variables, including disease severity and comorbid conditions, to affect patient outcomes. Consequently, prompt recognition and management of SAD in elderly patients is crucial to enhance their prognosis.

This study identified that within the XGBoost predictive model, coagulation parameters in patients with SAD, specifically PT and PTT, served as significant risk factors for predicting 30-day mortality in this patient population. PT and PTT have been recognized as significant prognostic markers in numerous clinical scenarios. In individuals with SAD, deviations in coagulation function may be strongly correlated with unfavorable outcomes. Research indicates that extended PT is associated with increased mortality rates among sepsis patients, potentially due to the activation of the coagulation cascade, resulting in multiple organ dysfunction.^[27]^ Abnormalities in PTT have been identified as independent prognostic factors for unfavorable outcomes in patients with sepsis.^[28]^ In the ICU, fluctuations in coagulation parameters are commonly used to evaluate the severity of illness and predict patient prognosis. Research has demonstrated a significant correlation between extended PT and PTT, with both in-hospital mortality and 1-year mortality rates among individuals with sepsis.^[27]^ Additionally, extended PTT has been linked to an increased 28-day mortality rate in patients with sepsis-associated acute kidney injury.^[28]^ In cases of sepsis-induced multiple organ dysfunction, alterations in the coagulation system are indicated by an extended PT and PTT, which can be valuable indicators for evaluating patient prognosis.^[29]^ Moreover, studies have shown that in individuals with sepsis, irregularities in coagulation processes are strongly linked to the dysregulation of the inflammatory response, potentially worsening the progression of the illness.^[30]^ In conclusion, evaluation of PT and PTT plays a crucial role in the prognostic assessment of patients with sepsis-related delirium. These parameters not only assist in identifying high-risk patients but also serve as a foundation for clinical interventions, ultimately enhancing patient outcomes.^[31,32]^

This study demonstrated that individuals with a prior stroke exhibit an elevated 30-day mortality rate, and a history of stroke serves as a significant risk factor for negative outcomes in patients with SAD. The correlation between prior stroke and SAD has been substantiated in numerous studies. Specifically, research suggests that a history of stroke is a critical risk factor for delirium onset in patients with sepsis. This vulnerability may stem from the compromised state of the nervous system in stroke survivors, rendering them more prone to inflammatory responses elicited by sepsis, which subsequently contributes to the development of delirium.^[33]^ A history of stroke not only elevates the risk of delirium in patients with sepsis, but also has a considerable effect on prognosis. Research indicates that septic patients with a history of stroke have a significantly increased likelihood of negative outcomes after developing delirium. This heightened risk may be linked to the generally poor baseline health of stroke survivors, rendering them more vulnerable to severe complications in the context of sepsis.^[1]^ Subsequent studies demonstrated that SAD significantly influences the long-term functional outcomes of patients. Additionally, a history of stroke may intensify the adverse effects of SAD on patient recovery, resulting in increased rates of mortality and disability.^[34]^ In clinical settings, recognizing and addressing the history of stroke is essential for evaluating the risk of delirium in patients with sepsis. By promptly identifying high-risk individuals, healthcare providers can apply more effective preventive measures and intervention strategies, ultimately enhancing patient outcomes.^[35]^ A history of stroke is an independent risk factor for the development of delirium in patients with sepsis and significantly influences patient outcomes. Consequently, it is crucial to prioritize the prevention and management of delirium in the care of patients with sepsis, particularly those with a history of stroke.^[36,37]^

This study indicates that reduced hemoglobin levels are significantly associated with unfavorable outcomes in individuals with SAD. Through a systematic review and meta-analysis, the investigators identified a notable association between hemoglobin concentrations and the prognosis of patients with sepsis, revealing that lower hemoglobin levels are associated with increased mortality rates.^[38]^ In investigations concerning SAD, a reduction in hemoglobin levels is recognized as a significant factor influencing unfavorable patient outcomes. Evidence suggests that low hemoglobin concentrations are directly linked to both the onset and duration of delirium, potentially resulting from inadequate oxygen delivery due to anemia, which subsequently affects cerebral function.^[39]^ Additionally, anemia may increase the risk of delirium by intensifying the systemic inflammatory responses and oxidative stress.^[40]^ In clinical settings, recognizing and addressing anemia is essential to enhance the outcomes of patients with sepsis. Studies indicate that hemoglobin levels in patients with sepsis should be regularly assessed, and timely interventions should be undertaken to mitigate the risk of delirium and other negative consequences.^[41,42]^ Moreover, prompt detection and management of anemia could potentially decrease the occurrence of SAD and enhance the overall outcomes of patients.^[43]^

## 5. Limitations

This study had several limitations. First, despite high sensitivity and AUROC, the low specificity (~0.1) of the models may limit their clinical utility by generating false positives. Future models should aim to improve specificity through feature engineering or alternative algorithms. Second, the data utilized in this research predominantly center on clinical biochemical indicators, comorbidities, and treatment approaches, while lacking a multimodal analysis of patient imaging data, such as cranial CT scans, cranial MRIs, and electroencephalograms. Consequently, this may introduce bias into the data analysis. Third, because the MIMIC-IV database is derived from the Beth Israel Deaconess Medical Center (BIDMC) in the United States, the model may reflect biases related to specific conditions. Therefore, when implementing this model in other healthcare settings, it is essential to either retrain the model or incorporate external validation using data from different hospitals to improve its accuracy.

## 6. Conclusions

This study developed a machine-learning model aimed at predicting 30-day mortality in patients with SAD, incorporating various risk factors including patient age, blood urea nitrogen levels, coagulation status, medical history, and anemia. The model exhibited strong accuracy and reliability after internal validation, indicating its potential for clinical implementation and dissemination. However, as a machine-learning model, it lacks interpretability, which is typically associated with traditional regression models. Moreover, there is the potential for enhancement by validating this predictive model against additional external datasets. The clinical risk factors integrated into this model do not include other forms of multimodal data such as CT scans, MRI, and EEG. Future investigations could integrate these additional data types to create a multimodal machine-learning predictive model and improve external validation using larger multicenter datasets, thereby enhancing the generalizability of this predictive tool.

## Acknowledgments

The authors thank all the participants for their contributions.

## Author contributions

**Data curation:** Jiancheng Zhang.

**Supervision:** Guangjun Jin.

**Writing – original draft:** Jingjing Yin, Xuming Pan.

**Writing – review & editing:** Danlei Chen.

## References

[1] Zheng G Yan J Li W Chen Z. Frailty as an independent risk factor for sepsis-associated delirium: a cohort study of 11,740 older adult ICU patients. Aging Clin Exp Res. 2025;37:52. 40011361 10.1007/s40520-025-02956-2 PMC11865144

[2] Zhang Y Hu J Hua T Zhang J Zhang Z Yang M. Development of a machine learning-based prediction model for sepsis-associated delirium in the intensive care unit. Sci Rep. 2023;13:12697. 37542106 10.1038/s41598-023-38650-4 PMC10403605

[3] Van Den Boogaard M Wassenaar A Van Haren FMP. Influence of sedation on delirium recognition in critically ill patients: a multinational cohort study. Aust Crit Care. 2020;33:420–5. 32035691 10.1016/j.aucc.2019.12.002

[4] Spiegelberg J Song H Pun B Webb P Boehm LM. Early identification of delirium in intensive care unit patients: improving the quality of care. Crit Care Nurse. 2020;40:33–43. 10.4037/ccn2020706 PMC7426069 32236428

[5] Von Hofen-Hohloch J Awissus C Fischer MM Michalski D Rumpf J-J Classen J. Delirium screening in neurocritical care and stroke unit patients: a pilot study on the influence of neurological deficits on CAM-ICU and ICDSC outcome. Neurocrit Care. 2020;33:708–17. 32198728 10.1007/s12028-020-00938-y PMC7736013

[6] Mușat F Păduraru DN Bolocan A. Machine learning models in sepsis outcome prediction for ICU patients: integrating routine laboratory tests—a systematic review. Biomedicines. 2024;12:2892–2892. 39767798 10.3390/biomedicines12122892 PMC11727033

[7] Zhang Y Xu W Yang P Zhang A. Machine learning for the prediction of sepsis-related death: a systematic review and meta-analysis. BMC Med Inform Decis Mak. 2023;23:283. 38082381 10.1186/s12911-023-02383-1 PMC10712076

[8] Ocagli H Bottigliengo D Lorenzoni G. A machine learning approach for investigating delirium as a multifactorial syndrome. Int J Environ Res Public Health. 2021;18:7105. 34281037 10.3390/ijerph18137105 PMC8297073

[9] Jauk S Kramer D Großauer B. Risk prediction of delirium in hospitalized patients using machine learning: an implementation and prospective evaluation study. J Am Med Inform Assoc. 2020;27:1383–92. 32968811 10.1093/jamia/ocaa113 PMC7647341

[10] Chen H Yu D Zhang J Li J. Machine learning for prediction of postoperative delirium in adult patients: a systematic review and meta-analysis. Clin Ther. 2024;46:1069–81. 39395856 10.1016/j.clinthera.2024.09.013

[11] Ke X Zhang F Huang G Wang A. Interpretable machine learning to optimize early in-hospital mortality prediction for elderly patients with sepsis: a discovery study. Comput Math Methods Med. 2022;2022:1–10. 10.1155/2022/4820464 PMC9779998 36570336

[12] He B Qiu Z. Development and validation of an interpretable machine learning for mortality prediction in patients with sepsis. Front Artif Intell. 2024;7:1348907. 39040922 10.3389/frai.2024.1348907 PMC11262051

[13] Shen X Shang D Sun W Ru S. Machine learning approach for the prediction of 30-day mortality in patients with sepsis-associated delirium. PLoS One. 2025;20:e0319519. 40202964 10.1371/journal.pone.0319519 PMC11981165

[14] Chen T Guestrin C. XGBoost: a scalable tree boosting system. In: Krishnapuram B Shah M Smola AJ Aggarwal C, eds. Proceedings of the 22nd ACM SIGKDD International Conference on Knowledge Discovery and Data Mining. ACM; 2016:785–94.

[15] Gu Q Yang S Fei D Lu Y Yu H. A nomogram for predicting sepsis-associated delirium: a retrospective study in MIMIC III. BMC Med Inform Decis Mak. 2023;23:184. 37715189 10.1186/s12911-023-02282-5 PMC10503010

[16] Yamamoto T Mizobata Y Kawazoe Y. Incidence, risk factors, and outcomes for sepsis-associated delirium in patients with mechanical ventilation: A sub-analysis of a multicenter randomized controlled trial. J Crit Care. 2020;56:140–4. 31901649 10.1016/j.jcrc.2019.12.018

[17] Deng T Wu D Liu SS Chen XL Zhao ZW Zhang LL. Association of blood urea nitrogen with 28-day mortality in critically ill patients: a multi-center retrospective study based on the eICU collaborative research database. PLoS One. 2025;20:e0317315. 39808678 10.1371/journal.pone.0317315 PMC11731709

[18] Ma A Zhang C Gong Y Ma X Yan N. The association between blood urea nitrogen to serum albumin ratio and 28 day in-hospital mortality in patients with chronic heart failure and sepsis: a pilot retrospective study. Front Cardiovasc Med. 2025;12:1491331. 40104144 10.3389/fcvm.2025.1491331 PMC11914131

[19] Fang Y Tang X Gao Y. Association between blood urea nitrogen and delirium in critically ill elderly patients without kidney diseases: a retrospective study and mendelian randomization analysis. CNS Neurosci Ther. 2025;31:e70201. 39754021 10.1111/cns.70201 PMC11702503

[20] Han T Cheng T Liao Y. Analysis of the value of the blood urea nitrogen to albumin ratio as a predictor of mortality in patients with sepsis. J Inflamm Res. 2022;15:1227–35. 35558187 10.2147/JIR.S356893 PMC9089193

[21] Wang Y Gao S Hong L. Prognostic impact of blood urea nitrogen to albumin ratio on patients with sepsis: a retrospective cohort study. Sci Rep. 2023;13:10013. 37340147 10.1038/s41598-023-37127-8 PMC10282077

[22] Park WR Kim HR Park JY Kim HE Cho J Oh J. Potential usefulness of blood urea nitrogen to creatinine ratio in the prediction and early detection of delirium motor subtype in the intensive care unit. J Clin Med. 2022;11:5073. 36078999 10.3390/jcm11175073 PMC9457387

[23] Liang JH Wang LM Song SF. Associations of the triglyceride-glucose index with mortality mediated by blood urea nitrogen among critically ill patients: a cohort study. Sci Rep. 2025;15:11149. 40169683 10.1038/s41598-024-83093-0 PMC11961709

[24] Lei W Ren Z Su J. Immunological risk factors for sepsis-associated delirium and mortality in ICU patients. Front Immunol. 2022;13:940779. 36203605 10.3389/fimmu.2022.940779 PMC9531264

[25] Xu C Lv L Hu W Yu Z Qian H Chen F. Long-term outcomes in older patients with sepsis in the ICU: a retrospective study. Altern Ther Health Med. 2024;30:124–30. 37856802

[26] Vasilevskis EE Han JH Hughes CG Ely EW. Epidemiology and risk factors for delirium across hospital settings. Best Pract Res Clin Anaesthesiol. 2012;26:277–87. 23040281 10.1016/j.bpa.2012.07.003 PMC3580997

[27] Zheng R Pan H Wang JF Yu X-S Chen Z-Q Pan J-Y. The association of coagulation indicators with in-hospital mortality and 1-year mortality of patients with sepsis at ICU admissions: a retrospective cohort study. Clin Chim Acta. 2020;504:109–18. 32044332 10.1016/j.cca.2020.02.007

[28] Lin C Wang J Cai K. Elevated activated partial thromboplastin time as a predictor of 28-day mortality in sepsis-associated acute kidney injury: a retrospective cohort analysis. Int J Gen Med. 2024;17:1739–53. 38706747 10.2147/IJGM.S459583 PMC11069355

[29] Guo QY Peng J Shan TC Xu M. Risk factors for mortality in critically ill patients with coagulation abnormalities: a retrospective cohort study. Curr Med Sci. 2024;44:912–22. 39285052 10.1007/s11596-024-2920-0

[30] Van Vught LA Uhel F Ding C. Consumptive coagulopathy is associated with a disturbed host response in patients with sepsis. J Thromb Haemost. 2021;19:1049–63. 33492719 10.1111/jth.15246 PMC8048632

[31] Zhang L Li X Huang J. Predictive model of risk factors for 28-day mortality in patients with sepsis or sepsis-associated delirium based on the MIMIC-IV database. Sci Rep. 2024;14:18751. 39138233 10.1038/s41598-024-69332-4 PMC11322336

[32] Chen P Ge Y Sheng H. The role of early changes in routine coagulation tests in predicting the occurrence and prognosis of sepsis. World J Emerg Med. 2025;16:136. 40135212 10.5847/wjem.j.1920-8642.2025.036 PMC11930565

[33] Lin C Zhang H Xiao F. Delirium is a potential predictor of unfavorable long-term functional outcomes in patients with acute ischemic stroke: a prospective observational study. J Inflamm Res. 2025;18:4019–35. 40125080 10.2147/JIR.S505038 PMC11929518

[34] Cui C Han G Wang Y Zhao B Li Q. Development and validation of a nomogram for predicting model for delirium after stroke. Asian Nurs Res. 2025;19:113–9. 10.1016/j.anr.2025.01.001 40010664

[35] Ketema B Mengistu G Melka D Zenebe Y Zebenigus M Leul F. A multicenter prospective study on the prevalence of post stroke delirium and associated risk factors in Addis Ababa, Ethiopia. BMC Neurol. 2025;25:114. 40108494 10.1186/s12883-025-04114-7 PMC11921746

[36] Zuo L Dong Y Hu Y. Heart failure, dementia is associated with increased stroke severity, in‐hospital mortality and complications. ESC Heart Fail. 2025;12:2066–76. 40128911 10.1002/ehf2.15216 PMC12055424

[37] Hahn M Brockstedt L Gröschel S. Delirium following mechanical thrombectomy for ischemic stroke – individuals at risk, imaging biomarkers and prognosis. Front Aging Neurosci. 2025;17:1486726. 40026421 10.3389/fnagi.2025.1486726 PMC11868270

[38] Zhu J Dong Y Liao P Yin X He J Guo L. Prognostic value of hemoglobin in patients with sepsis: a systematic review and meta-analysis. Heart Lung. 2023;64:93–9. 38070279 10.1016/j.hrtlng.2023.12.001

[39] Boran Y Altunci YA Yalçinli S. Mortality-related factors in older adults with delirium: a prospective observational study. Geriatr Nurs. 2024;60:427–32. 39413553 10.1016/j.gerinurse.2024.10.005

[40] Yao P Wu L Yao H Shen W Hu P. Acute hyperglycemia exacerbates neuroinflammation and cognitive impairment in sepsis-associated encephalopathy by mediating the ChREBP/HIF-1α pathway. Eur J Med Res. 2024;29:546. 39538358 10.1186/s40001-024-02129-3 PMC11562611

[41] Hou L Wu X Sun Z. Risk factors and prognosis of acute kidney injury in hospitalised sepsis patients. Arch Esp Urol. 2024;77:263–9. 38715167 10.56434/j.arch.esp.urol.20247703.35

[42] Zhu YB Liu TL Dai Q. Characteristics and risk factors for pediatric sepsis. Curr Med Sci. 2024;44:648–56. 38748371 10.1007/s11596-024-2870-6

[43] Fang Y Dou A Shen Y. Association of triglyceride-glucose index and delirium in patients with sepsis: a retrospective study. Lipids Health Dis. 2024;23:227. 39054513 10.1186/s12944-024-02213-x PMC11271053

